# ‘No right time, no right place’ – conversations in acute cancer care – results of a Macmillan focus group

**DOI:** 10.1016/j.fhj.2025.100246

**Published:** 2025-03-29

**Authors:** Lillis Ashling, Williams Hilary, Marshall Ernie, Jones Michael, Henderson Sophie, Minton Ollie

**Affiliations:** a Macmillan Cancer Support, London, UK; b Velindre Cancer Centre, Cardiff, UK; c Clatterbridge Cancer Centre, Liverpool, UK; d University Hospitals Sussex, Brighton, UK

## Abstract

**Background:**

We present work funded by Macmillan Cancer Support and hosted with the UK Acute Oncology Society from conversations about acute cancer care with oncologists, palliative care and generalist clinicians to determine how best to manage the increasing complexity of cancer patients and their management.

**Methods:**

We used a number of facilitated focus groups to draw out themes of initial management, communication differentiating between toxicity, disease progression and when and how to start conversations about the end of life.

**Results:**

There is an awareness of the significant mortality for patients needing acute cancer care. However, there is no clear responsibility to having these prognostic conversations. These include attitudes towards future care planning (FCP) in oncology, ownership and the challenges faced by non-oncologists treating more and more people living with cancer.

**Conclusions:**

There needs to be increased recognition of acute admission as a point of transition, often into end-of-life care, for people with cancer. There are significant barriers to conversations in acute cancer care, which include a lack of clear ownership in oncology pathways, and a reticence of all professionals to engage in conversations around prognosis before acute admission or at the time of acute illness. There is a need to develop standardised links between oncology and urgent care providers and for protocols within hospital to allow for access to expertise and assessment and onward sharing of information. Earlier, more transparent conversations may allow patients and families to make clearer choices about care options in the last months of life and, critically, reduce dependence on acute care.

## Introduction

Unplanned cancer care is a significant issue for the NHS and there is a growing number of patients who have unplanned contact with the hospital, usually in the last years of life.^1^

The focus nationally is on time to treatment and diagnostic pathways^2^ and so, by default, there is a disconnect between elective outpatient care and emergency care, frequently provided by a clinical team who are geographically separate from the treating clinicians. Many healthcare professionals are honest about how difficult they find talking about end of life with patients because of the complexity of cancer treatment^4^ and time pressures of service delivery. Some healthcare professionals state that the end-of-life conversation can be made more difficult because there is a lack of communication training.^5^^,^^6^

Previous work in this area^1^^,^^3^ has consistently shown a poor prognosis for acute admissions. We have shown that 20% of all acute oncology contacts have died within 30 days and 70% at 12 months.^1^^,^^3^ However, within this there are a population with better prognosis and who need to have access to the necessary oncology, supportive and palliative care services.^7^, ^8^, ^9^

Our aim of initiating these focus groups derived from the need to address this uncertainty at the time of a non-elective contact with the hospital and to explore whose responsibility is this when the majority of acute cancer care is provided by non-oncologists.^7^, ^8^, ^9^ We also wanted to explore the best make-up of these teams and identify the skills and learning required to look after all cancer patients. We wanted to explore in more depth attitudes to future care planning (FCP) and why these conversations are not happening on a more regular basis.

## Methods

We convened four focus groups through purposive sampling and national advertising. All participants gave written consent to participate and an agreement that aggregated data could be shared anonymously, including for publication. As this was solely a service improvement project, under the Health Research Authority (HRA) criteria we did not apply for ethics committee approvals, but used internal Macmillan governance process.

The focus groups were with oncologists and non-oncology healthcare professionals (90 min each), all working in the UK NHS. In each group the same questions were posed and discussions focused on attitudes, triggers, barriers and solutions to having FCP conversations more regularly.

The different sessions were segregated into two for oncologists (consultants and senior resident doctors) and two for non-oncology healthcare professionals (including clinical nurse specialists) who meet acutely unwell cancer patients. Each group was facilitated by a trained expert in focus group facilitation commissioned by Macmillan and all group discussions were recorded and transcribed and themes collated.

## Results

We found recurring themes around timings, therapeutic optimism and confidence in having these conversations. The overarching theme from the oncologists is around ‘ownership of treatment pathways’ and maintaining a therapeutic relationship. Oncologists noted that patients wanted to ‘fight their cancer’ and by discussing the future trajectory of the illness, patients may ‘lose hope’ and there was a ‘joint unspoken bond’ that finding another treatment option was the focus of the clinic appointment.

The non-oncologists appeared to have more confidence and understanding of whole-system care.

The themes are detailed in Table 1 (the difference between oncologists and non-oncologists) and Table 2 (there is no right time or right place to have these conversations). From the focus groups, there are also some illustrative quotes not attributed to individuals below.

**Table 1:** Approach between oncologists and non-oncologists.

Timing of conversation	Place of conversation	Confidence and approach	Specific cancer language and expertise
Most oncologists said that they felt they do have the responsibility to have FCP discussions with patients during an acute admission and they do so when ‘it is appropriate.’ Non-oncologists conversely said that conversations around FCP were not commonly undertaken by oncology during acute admissions.	Acute settings not seen as the right time or place as family are often not present, the patient is unwell and the treating oncologist is not present. This leads to further delays as the conversation is deferred to a ‘future time’, which often doesn’t happen. Healthcare professionals said that conversations take place ‘too late’ – and this can mean that patients become distressed at why the conversation wasn’t had earlier.	Oncologists spoke more about the fear of ‘*getting it wrong*’. This is driven both by the nature of the topic itself, but also due to past adverse experiences. Oncologists were open and honest about the knowledge that some of their colleagues won’t/don’t have these conversations, either due to a lack of skills or a tendency to focus on treatment.	Oncology services tend to propagate the narrative that healthcare professionals are meant to be consistently seen to be ‘fighting cancer’ on behalf of patients. This close relationship between patient and oncologist reinforces the issues around patient ownership, but is valuable to people living with cancer.
Non-oncologists don’t always feel confident in having the conversation as they don’t perceive themselves to be the ‘experts’ in cancer care, especially with many novel cancer treatments. Oncologists expressed concern that non-cancer colleagues can be too pessimistic about prognosis and can fail to interpret cancer-specific information.			Oncologists want to focus on the immediate issue and potential new lines of treatment.

**Table 2:** There is no right time, no right place to have these conversations.

Confidence and approach	Toxic optimism	Triggers for conversations	Oncology exceptionalism
It’s seen as a ‘difficult’ conversation, oncology professionals expressed more anxiety and worry about ‘getting it wrong’, non-oncology professionals were much more positive about these conversations.	Oncologists tended to overestimate 12-month prognosis in those people who have acute admissions. They discussed that they tended not to share prognostic information in communications to patients/other HCPs, even when they ‘know’ it is likely not to ‘go well’ during anti-cancer treatment.	Oncologists did not see an acute admission in itself as a trigger for FCP in the same way as non-oncology HCPs did. Disease progression on scans and lack of further lines of available treatment were perceived as much stronger triggers. Non-oncologists felt comfortable that an acute admission was enough of a reason for an FCP conversation.	Oncology is viewed as ‘different’ from other diseases by patients and professionals. The geographical separation of cancer services and the cultural attitude to ‘fighting’ cancer reinforce this.

‘Part of it is that you don’t want to be seen as the bad guy … you’re delivering bad news to them and this can change the relationship.’ Oncologist FG1

‘It’s not a nice conversation to have. If there is further treatment that you’re planning on giving … if they seem sort of relatively well at the time … then we don’t need to talk about it right now.’ Oncologist FG4

‘It’s going to take us a long time to fix this. So, in the meantime, we should be passing ownership to the patients themselves. Let’s give them the confidence to bring up conversations about end of life.’ Palliative care CNS FG3

## Discussion

We have added some qualitative professional data to our work on the wider healthcare use of cancer patients attending as an emergency^1^ and outlined some of the reasons this doesn’t trigger specific prognostic and end-of-life conversations. We have found a disconnect between the patient and family understanding of aims of anti-cancer treatment and the likely prognosis when patients present as an emergency. For non-oncologists, the admission may be seen as a key opportunity to support conversations. However, for both groups there is a concern with the increasing number and complexity of effective treatments, more intensive clinical intervention may be warranted.

The development of acute oncology services has aimed to provide better integration between treating and acute teams but there remain barriers to cross-sector working. Cancer outcomes are broadly focused and funded on time to treatment, and new effective treatments are funded for the drug costs and potentially the delivery costs, but not the costs of providing predictable management of toxicity. As an oncologist, it is easier to access high-cost drugs than to access funded community supportive care, and the very rapid growth of new drugs places the additional challenge of adequate workforce capacity to provide treatment and time to provide holistic care. This may improve as the curriculum for both medical and clinical oncology includes training in acute oncology.^10^

There are clear limitations to our study with our purposive sampling and exploratory nature. However, all sessions were conducted by a trained professional with extensive experience to maximise the generalisability of the themes, but these are not exhaustive. We can only extrapolate these findings with further work and feedback from the quantitative data^1^ to encourage local quality improvement work, training and service design. We only conducted this from a professional viewpoint, but would emphasise the importance of shared decision making in any conversation with cancer patients.

These non-elective hospital episodes are associated with high readmission rates.^1^ An acute admission must be viewed beyond crisis management, as an opportunity to consider temporary or permanent changes in patient needs, with effective communication of any changes or plans between GP and treating teams. Our ambition from this work and from Macmillan Cancer Support and other cancer charities is: Every person with cancer who has an unplanned acute admission has an opportunity for a personalised care planning conversation and care plan completed.

## Conclusions

Acute oncology episodes often reflect key moments of ‘transitions of care’, ie from curative to palliative treatment, either through disease progression or unmanageable toxicities. Such admissions therefore present an opportunity to support serious illness conversations. Given the short life expectancy of cancer patients presenting with acute needs, efforts should be focused on helping patients and families understand their future care options, enhancing quality of life for this cohort, supporting admission avoidance through adequate community response and ambulatory options to reduce the amount of time spent in hospital.

## Ethical approval

Not applicable. This is a service improvement study and written informed consent was obtained from all participants.

## CRediT authorship contribution statement

AL conception and writing OM writing SH MJ data collection all authors approved the final version.

## Declaration of competing interest

The authors declare the following financial interests/personal relationships which may be considered as potential competing interests: Associate editor for *Future Healthcare Journal* Williams H. If there are other authors, they declare that they have no known competing financial interests or personal relationships that could have appeared to influence the work reported in this paper.

## Data Availability

The data that support the findings of this study are available from the corresponding author upon reasonable request.
